# Draft Genome Sequences of Mycolicibacter senuensis Isolate GF74 and Mycobacterium colombiense Isolates GF28 and GF76 from a Swine Farm in Japan

**DOI:** 10.1128/MRA.00936-18

**Published:** 2018-09-13

**Authors:** Toshihiro Ito, Kotaro Sawai, Mikihiko Kawai, Keiko Nozaki, Keiko Otsu, Hideto Fukushi, Kenji Ohya, Fumito Maruyama

**Affiliations:** a Department of Microbiology, Graduate School of Medicine, Kyoto University, Sakyo-ku, Kyoto, Japan; b Laboratory of Veterinary Microbiology, Faculty of Applied Biological Sciences, Gifu University, Gifu, Japan; c Gifu Prefectural Chuo Livestock Hygiene Service Center, Gifu, Japan; d United Graduate School of Veterinary Sciences, Gifu University, Gifu, Japan; e Scientific and Technological Bioresource Nucleus, Universidad de La Frontera, Temuco, Chile; University of California, Riverside

## Abstract

Several nontuberculous mycobacteria (NTM) occasionally infect humans and animals. Here, we report the draft genome sequences of Mycolicibacter senuensis isolate GF74 (4,792,997 bp) and Mycobacterium colombiense isolates GF28 and GF76 (5,473,554 bp and 5,426,852 bp, respectively) isolated from a swine farm in Japan. These sequences provide further information on NTM research.

## Abstract (precis)

Several nontuberculous mycobacteria (NTM) occasionally infect humans and animals. Here, we report the draft genome sequences of Mycolicibacter senuensis isolate GF74 (4,792,997 bp) and Mycobacterium colombiense isolates GF28 and GF76 (5,473,554 bp and 5,426,852 bp, respectively) isolated from a swine farm in Japan.

## ANNOUNCEMENT

Nontuberculous mycobacteria (NTM), encompassing mycobacteria other than Mycobacterium tuberculosis complex and Mycobacterium leprae, include more than 170 species. NTM usually inhabit the natural environment, but most are considered opportunistic pathogens of humans and animals (1). Based on comprehensive phylogenomic analyses, it has been proposed that the single genus Mycobacterium be divided into five distinct monophyletic clades, as follows: an emended genus Mycobacterium (“Tuberculosis-Simiae” clade) and four novel genera, Mycolicibacterium gen. nov. (“Fortuitum-Vaccae” clade), Mycolicibacter gen. nov. (“Terrae” clade), Mycolicibacillus gen. nov. (“Triviale” clade), and Mycobacteroides gen. nov. (“Abscessus-Chelonae” clade) (2).

Recent comprehensive genomic studies have increased our knowledge on the genetic features and classification of NTM (2–4). However, more information is needed about NTM, including genome sequences, which are indispensable for understanding ecology and etiology and for developing reliable diagnostic tools. Here, we report the draft genome sequences of Mycolicibacter senuensis (basonym: Mycobacterium senuense) isolate GF74 and Mycobacterium colombiense isolates GF28 and GF76 from soil in Japan. Mycolicibacter senuensis, first isolated from a Korean patient with a symptomatic pulmonary infection, belongs to the Terrae clade (5). Mycobacterium colombiense, initially isolated from HIV-positive patients in Colombia, is a member of the Mycobacterium avium complex (6).

All isolates were obtained from mud at a swine farm in the Tokai area of Japan, as described previously (7). Briefly, the mud samples were decontaminated with equal volumes of 2% NaOH and then inoculated onto a 2% Ogawa slant (Kyokuto Pharmaceutical, Tokyo, Japan) at 37°C for up to 4 weeks. Each single colony on the slant was subcultured on Middlebrook 7H11 agar supplemented with 10% oleic acid-albumin-dextrose-catalase (OADC) enrichment (Becton, Dickinson, MD, USA). The species of the isolates were identified by analyzing 16S rRNA, *hsp65*, and *rpoB* genes (8, 9). DNA was extracted using a PureLink genomic DNA extraction kit, according to the manufacturer’s instructions (Invitrogen, Carlsbad, CA, USA), and paired-end libraries with an average insert size of 350 bp were prepared from each 3 µg of genomic DNA. These underwent 2 × 150-bp sequencing on a HiSeq X Ten sequencing platform (Illumina, San Diego, CA, USA) at the Beijing Genomics Institute (Shenzhen, China). Quality trimming and adapter trimming were conducted using Cutadapt (https://github.com/marcelm/cutadapt/) via TrimGalore! (https://github.com/FelixKrueger/TrimGalore). Mismatch correction of reads and assembly were carried out using SPAdes (10), and the assembly was polished using Pilon (11), with the aid of Unicycler (12). CheckM was used to estimate genome completeness (13). Draft genomes were then annotated using the NCBI Prokaryotic Genome Annotation Pipeline (PGAP) (14). The combined lengths of the final draft genomes, G+C contents, and the numbers of scaffolds, coding sequences (CDSs), rRNAs, and tRNAs are shown in Table 1. ANItools analysis (15) revealed that Mycolicibacter senuensis GF74 and Mycobacterium colombiense GF28 and GF76 showed 93.12% identity to Mycobacterium sp. strain JDM601, 86.73% identity to Mycobacterium indicus pranii, and 86.18% identity to Mycobacterium intracellulare MOTT, respectively.

**TABLE 1:** Summary information for the draft genome sequences of nontuberculous mycobacterial isolates obtained from mud at a swine farm

Species	Isolate	Genome size (bp)	No. of scaffolds^*a*^	G+C content (%)	No. of CDSs^*b*^	No. of rRNAs	No. of tRNAs	GenBank accession no.
Mycolicibacter senuensis	GF74	4,792,997	304	67.95	4,809	3	42	QMEX00000000
Mycobacterium colombiense	GF28	5,473,554	146	67.69	5,238	3	51	QMEV00000000
Mycobacterium colombiense	GF76	5,426,852	216	67.64	5,249	3	47	QMEU00000000

a Numbers of scaffolds >500 bp are shown.

b CDSs, coding sequences.

### Data availability.

The draft genome sequences of Mycolicibacter senuensis GF74 and Mycobacterium colombiense GF28 and GF76 have been deposited in DDBJ/EMBL/GenBank under the accession numbers QMEX00000000, QMEV00000000, and QMEU00000000, respectively (Table 1).
